# Older Adults With HIV Displayed Advantages in Weathering the Pandemic

**DOI:** 10.1093/geroni/igaf122.943

**Published:** 2025-12-31

**Authors:** Jennifer Kaufman, Yiyi Wu, Jasmine Manalel, Alvin Gao, Luis Scaccabarrozzi, Vera Antonios, Jerome Ernst, Mark Brennan Ing

**Affiliations:** Brookdale Center for Healthy Aging at Hunter College, New York, New York, United States; Hunter College, New York, New York, United States; Hunter College, CUNY, New York, North Carolina, United States; Hunter College, New York, New York, United States; Amida Care, New York, New York, United States; Amida Care, New York, New York, United States; Amida Care, New York, New York, United States; Hunter College, New York, New York, United States

## Abstract

The COVID-19 pandemic disrupted health care access, challenging care engagement among people living with HIV (PLWH). The pandemic was also a time when social connections frayed, threatening emotional well-being. As part of a qualitative study on care experiences of PLWH during the pandemic, we examined the differences between older and younger participants. We recruited 40 PLWH, aged 23-64, through a Medicaid managed care plan in New York City; 58% were 50 or older. Half the participants were non-Hispanic Black and 28% were Hispanic/Latinx; 55% were cisgender men, 25% cisgender women, and 20% transgender/nonbinary. Almost all the cisgender women were 50 or older. Average time since HIV diagnosis was 22 years. We conducted semistructured phone interviews during fall 2023 regarding the pandemic’s impact on care engagement, community program participation, and health and well-being. Overall, participants aged 50 and older reported strong bonds and often long-term relationships with their doctors and health care teams, as well as active community participation. They also expressed relative emotional stability during the pandemic, as behavioral health conditions were fairly well controlled. Participants in their 30s and 40s, by contrast, frequently reported experiences with anxiety, depression, and other behavioral health concerns, often coinciding with lower trust in the health care system. Building physician-patient trust may enhance both care engagement and emotional well-being. On the medical side, older adults, who have more comorbid conditions, reported difficulty seeing specialists and expressed an appreciation for coordinated care. These findings highlight the growing importance of care coordination as PLWH age.

